# Adverse childhood experiences and unhealthy dietary behaviours in adulthood

**DOI:** 10.1017/S1368980024000144

**Published:** 2024-01-18

**Authors:** Alexander Testa, Lixia Zhang, Dylan B Jackson, Kyle T Ganson, Julia H Raney, Jason M Nagata

**Affiliations:** 1 University of Texas Health Science Center at Houston, Department of Management, Policy and Community Health, 7411 John Smith Dr #1100, San Antonio, TX 78229, USA; 2 University of Louisville, Louisville, USA; 3 Johns Hopkins Bloomberg School of Public Health, Baltimore, USA; 4 University of Toronto, Toronto, Canada; 5 University of California, San Francisco, USA

**Keywords:** Adverse childhood experiences, Unhealthy dietary behaviour, Life course, Nutrition, Add health

## Abstract

**Objective:**

This study assesses the relationship between adverse childhood experiences (ACE) occurring before the age of 18 years and patterns of fast-food consumption and sugary beverage consumption in adulthood. The study also examines how perceived stress and socio-economic status (SES) (college educational attainment and income) in adulthood mediate this relationship.

**Design:**

Using data from the National Longitudinal Study of Adolescent to Adulthood Health (*N* 8599), multinomial logistic regression analyses were carried out to assess the association between ACE and unhealthy dietary behaviours in adulthood. Karlson–Holm–Breen mediation analysis is used to determine the mediating effects of SES and perceived stress.

**Setting:**

Persons living in the USA in 2016–2018.

**Participants:**

Adults (*n* 8599) aged 33–44 years.

**Results:**

The findings show an association between four or more ACE and high fast-food (relative risk ratio (RRR) = 1·436, 95 % CI = 1·040, 1·983) and high sugary beverage consumption (RRR = 1·435, 95 % CI = 1·002, 2·055). The association between ACE and high fast-food consumption is partially mediated by college educational attainment, and the association between ACE and high sugary beverage consumption is partially mediated by perceived stress and college educational attainment.

**Conclusions:**

ACE can have long-term consequences for unhealthy dietary behaviours in adulthood, and this relationship is partially due to a lower likelihood of higher perceived stress and college educational attainment among ACE-exposed persons. Future research is needed to understand further the influence of ACE on dietary patterns over the life course.

Overconsumption of unhealthy food items, including fast-food and sugary beverages, is a pressing public health concern. Indeed, the consumption of fast-food and sugary beverages is common among US adults, with over one-third regularly eating fast-food^(1)^ and over a quarter of adults consuming sugar-sweetened beverages^(2)^. Research underscores a connection between fast-food consumption and heightened risks of obesity, CVD and metabolic disorders^(3,4)^, and elevated sugary beverage intake is a risk factor for obesity and type 2 diabetes^(5,6)^.

Accordingly, understanding factors influencing the consumption of unhealthy food items, including fast-food and sugary beverages, is essential for health promotion efforts. Even so, the influence of exposure to early life adversity in childhood and adolescence as a potential risk factor for unhealthy dietary behaviour in adulthood remains under-studied. Potential risk factors for unhealthy dietary behaviour in adulthood are adverse childhood experiences (ACE) – that is, exposure to abuse, neglect and household dysfunction occurring before age 18 years old^(7)^. Despite robust evidence for the health consequences of ACE^(8)^, there is limited research on the long-term association between ACE and dietary behaviour. Commenting on the state of this literature, scholars have noted that ‘childhood trauma has an important but poorly understood relationship with diet^(9)^’. Importantly, the relationship between ACE and adult dietary behaviour may operate in a dose–response fashion such that greater ACE exposure corresponds with more fast-food and sugary beverage consumption. Having experienced four or more ACE is a critical threshold associated with a significantly elevated risk of poor health outcomes, as it amplifies the cumulative and synergistic impact of early-life adversities on physical and mental well-being^(8)^. Accordingly, it is possible that high ACE exposure is associated with higher fast-food and sugary beverage consumption levels in adulthood, considering that various forms of childhood trauma and adversity are related to poor dietary behaviour in childhood^(10,11)^, and research shows that dietary patterns in childhood inform nutritional behaviour in adulthood^(12)^.

One potential pathway for explaining why ACE are related to higher fast-food and sugary beverage consumption in adulthood is higher perceived stress. ACE are highly stressful events that exhibit connections to high levels of stress, which persist into adulthood^(13)^. Stress sensitisation theory proposes that repeated stress early in life results in a dysregulation of stress response systems and neurophysiological sensitivity, which lowers the threshold for adaptive responses over the life course and contributes to dysfunctional coping strategies in the aftermath of a stressful experience^(13)^. Stress also plays a vital role in dietary behaviour, leading individuals to select more palatable, gratifying and calorie-dense foods as a coping strategy or due to stress-related disinhibition^(14)^. Research has found that stress contributes to the overconsumption of high-calorie comfort foods, including fast-food, snacks and sugar consumption^(15,16)^. Prior studies have also theorised a possible connection between ACE and adult obesity due to stress-induced cravings for palatable foods^(17)^.

A second potential pathway explaining the relationship between ACE and adult nutrition can operate through an individual’s socio-economic status (SES). While an individual’s family childhood SES is associated with ACE exposure^(18)^, early life adversity is also disruptive to life-course trajectories and contributes to accumulating disadvantage that negatively influences an individual’s adult SES, particularly educational attainment and income levels^(19)^. According to the cumulative disadvantage framework, because ACE occur during a developmentally important period, they are salient life events that can be particularly consequential for socio-economic trajectories in adulthood^(20)^. A previous study finds that the relationship between ACE and adult health partially occurs through one’s adult SES^(21)^. A distinct area of research documents that higher SES is associated with healthier dietary patterns, whereas lower SES persons are more likely to have unhealthier dietary behaviours^(22)^. While individual-level SES is a multi-dimensional concept, specific features, such as high educational attainment and higher income, appear to be strongly connected with a healthier diet (i.e. greater consumption of fruits, vegetables and lean protein)^(23)^. Of notable importance is emerging research in recent years that demonstrates a stark educational divide in health and health behaviours between those with and without a college education^(24,25)^, with scholars contending that ‘of the components of socioeconomic status, education is particularly important because it has the strongest relationship to health and is generally established earlier in the life course^(24)^’.

Despite the possible direct relationship between ACE and unhealthy dietary behaviours and indirect relationships through stress and SES pathways, it appears only a few studies to date have come close to explicitly investigating these relationships. Among studies focused on children, studies have found a relationship between greater ACE exposure and poorer diet quality (lower intake of daily intake of fruits and vegetables, and greater consumption of sweet snacks, and sugar-sweetened beverages) via parent reports among young children at age 4 years old using data from the Early Childhood Longitudinal Study – Birth Cohort, collected in years 2005–2006^(11)^ or 10–11 years old from children who participated in the Adolescent Brain and Cognitive Development Study in years 2017–2019^(10)^. Among studies focused on adults, Aquilina and colleagues^(9)^ assessed the relationship between ACE and adult diet quality using data from a FFQ of eighty-nine different foods using a sample of low-income adults (aged 40–79) from the Southeast United States. The results showed that a higher ACE score was associated with poorer diet quality after converting responses – which do not include fast-food or sugary beverage consumption – into the Healthy-Eating Index-2010 (HEI-10). Outside the USA, research by Bellis and colleagues^(26)^ of 771 households in England found 13·6 % of the variation in poor diet (measured by whether a respondent had less than two portions of fruit and vegetable intake on a typical day) was due to ACE exposure. Notwithstanding the abovementioned research’s contributions, no studies have focused on how accumulating exposure to ACE influences fast-food and sugary beverage consumption levels in adulthood. Furthermore, no studies have assessed whether the relationship between ACE and unhealthy dietary behaviour is explained by perceived stress and SES.

The present study seeks to address gaps in the existing research via the following two aims. Our first aim is to assess whether accumulating ACE exposure is associated with poorer dietary behaviour measured by levels of fast-food consumption and sugary beverage consumption in middle adulthood (aged 33–44). Our second aim is to test the extent to which perceived stress and two indicators of adult SES (i.e. college educational attainment and income) mediate the relationship between ACE and unhealthy dietary behaviours in adulthood.

## Data

### Study setting and population

The data used in the current study are from the National Longitudinal Study of Adolescent to Adult Health (Add Health). The Add Health study is a nationally representative longitudinal study of adolescents enrolled in grades 7–12 in public, private and parochial schools in the USA in the 1994–1995 academic year (Wave I; aged 12–19 years). The initial sampling frame for the Add Health study was based on the Quality Education Database, which includes 26 666 high schools across the USA, from which a stratified sampling method was used to select eighty high schools, defined as institutions with an 11th grade and a student population exceeding 30. The selection probability was adjusted in accordance with the size of each school. The stratification criteria encompassed geographical region, urban or rural setting, type of school (such as public, private or parochial), ethnic diversity and overall school size. Additionally, for each high school chosen, the Add Health research team systematically identified and involved one feeder school, typically a middle school. The likelihood of a feeder school being selected was based on the number of students it contributed to the respective high school. This process resulted in eighty unique pairs of schools, each representing a different community. Initially, over 70 % of the selected schools consented to participate. To reach the target sample size, alternate schools were continually selected within each stratum as needed. Ultimately, 79 % of the schools approached agreed to participate in the Add Health study. The final sample comprised eighty high schools and fifty-two middle schools^(27)^.

Since the initial wave, there have been four follow-up periods of data collected: Wave II (1995–1996; aged 13–20 years; response rate 88·6 %), Wave III (2001; aged 18–26 years; response rate 77·4 %), Wave IV (2008–2009; aged 24–32 years; response rate 80·3 %) and Wave V (2016–2018; aged 33–44 years; response rate 71·8 %). Add Health uses multiple methods and techniques for panel maintenance and tracing to locate and schedule an interview with eligible respondents, including those who did not respond in a prior wave^(28)^.

### Survey administration

At each wave, respondents completed a survey questionnaire about various demographic, social, familial, socio-economic, behavioural, psychosocial, cognitive and health information. Responses to the survey were self-reported using computer-assisted self-interview technology. Surveys included questions on experiences of ACE asked at Waves I, III and IV, and patterns of fast-food and sugary beverage consumption at Wave V.

### Analytic sample

The analytic sample comprised respondents who participated in Waves I, III, IV and V. These waves were selected because the outcome and mediating variables were measured at Wave V, and measures used to construct the ACE measure were from Waves I, III and IV.

Missing data on ACE and control and mediator variables were addressed using multiple imputations with chained analysis in STATA 17 using the ‘mi impute’ command, resulting in twenty multiple imputed datasets (*n* 8599). Multiple imputation operates by creating several complete datasets, imputing missing values in a variable-by-variable sequence using the remaining variables as predictors in a regression framework. This imputation process, iteratively conducted across different variables with missing values, harnesses the entire dataset to inform the imputation model, thereby preserving the inherent relationships and patterns within the data. By generating multiple imputed datasets, multiple imputation often increases efficiency and reduces bias in estimates compared with listwise deletion under several conditions of missingness^(29,30)^.

### Ethics approval

The University of Texas Health Science Center at Houston Institutional Review Board approved the use of Add Health data for this study.

## Survey measures

### Dependent variables

*Fast-food consumption* was based on respondent self-reports from a question at Wave V asking, ‘In the past 7 days, how many times did you eat food from a fast-food restaurant, such as McDonald’s, Burger King, Wendy’s, Arby’s, Pizza Hut, Taco Bell, or Kentucky Fried Chicken or a local fast-food restaurant?’ *Sugary beverage consumption* was based on self-reports from a question at Wave V asking, ‘In the past 7 days, how many regular (non-diet) sweetened drinks did you have? Include regular soda, juice drinks, sweetened tea or coffee, energy drinks, flavored water, or other sweetened drinks^(31)^’.

To better capture consumption levels, particularly high consumption of fast-food and sugary beverages, while ensuring adequate cell sizes for statistical analyses, each measure was classified into quartiles using the *xtile* command in STATA, which uses the existing data distribution to create a variable containing quantile categories. Thus, we generate cutpoints based on the distribution of each variable, given the lack of guidance on appropriate cutpoints in prior research. For the fast-food consumption measure, respondents are classified into: *none* (zero times per week), *low* (1 time per week), *moderate* (2–3 times per week) and *high* (4 or more times per week). For the sugary beverage consumption measure, respondents are classified into *very low* (0–1 times per week), *low* (2–4 times per week), *moderate* (5–7 times per week) and *high* (8 or more times per week).

### Independent variable

Consistent with the procedure used in prior research with Add Health data^(32)^, the *ACE* variable was created as follows. First, ten variables were constructed based on survey responses indicating whether a respondent had no exposure (0) or exposure (1) to the following ACE: emotional abuse, physical abuse, sexual abuse, community violence, substance abuse, suicide, divorce, parental incarceration, emotional neglect and physical neglect. Next, the ten binary indicators were summed into a cumulative ACE index, which was used to create a categorical count of ACE, including 0, 1, 2, 3 or 4 or more ACE. ACE were top-coded at four as prior research finds this to be a threshold at which there is a particularly heightened risk for adverse health-related outcomes^(8,33)^. Appendix A details the questions used to construct each ACE item, the coding scheme, the survey wave used and the prevalence of each ACE measure in the final analytic sample.

### Mediating variables: perceived stress

*Perceived stress* was measured using a scale based on the following four items derived from the validated Perceived Stress Scale^(34)^. Respondents were asked in the past 30 d: (1) how often have you felt that you were unable to control the important things in your life, (2) how often have you felt confident in your ability to handle your personal problems (reverse coded), (3) how often have you felt things were going your way (reverse coded) and (4) how often have you felt that difficulties were piling up so high that you could not overcome them. Response options included: never, almost never, sometimes, fairly often and very often. Responses were summed into a single index (range 0–16) where higher scores equate to higher perceived stress (*α* = 0·787).

### Mediating variables: socio-economic status

*College educational attainment* is a binary variable measuring whether a respondent reported graduating by Wave V. The variable is measured by asking respondents ‘What is the highest level of education that you have achieved to this date’. Respondents who answered completed a 4-year college degree or higher (i.e. ‘bachelor’s degree’, ‘some graduate school’, ‘completed a master’s degree’, ‘completed a doctoral degree’ or ‘some post-baccalaureate professional education’) were coded as having college educational attainment. Respondents who answered that having an educational attainment was less than a bachelor’s degree were coded as not having college educational attainment.

*Adjusted household income* measured the total household income while adjusting for the number of people living in a household. At Wave V, respondents were asked about the total household income before taxes and deductions. Income was reported using intervals ranging from less than $5000 to $200 000 or more. Consistent with prior Add Health research, the mid-point of each response category was used to generate a continuous measure of income.^(35)^ Consistent with procedures in prior Add Health research, household income was adjusted for the number of people living in the home using the equivalence of scale method, where adjusted household income = household income/(household size) × 0·5. Income was log-transformed to reduce positive skew^(36)^.

### Control variables

Control variables included *age* in years at Wave V, respondent’s *race/ethnicity* (White, Black, Hispanic, Other race/ethnicity), *biological sex* (1 = male, 0 = female), *born in the US* (1 = yes, 0 = no), SES status from Wave I was derived from a preconstructed variable, which is a *z*-transformed factor score of reports from Wave I based on parental education, parental occupation, household income and household receipt of public assistance^(37)^, the number of time a respondent reported eating (a) *fruit* or (b) *vegetables* the day before their Wave I interview (0 = none, 1 = once, 2 = more than once), *currently married* (1 = yes, 0 = no) and *depressive symptoms* at Wave I were based on items from the validated Centers for Epidemiological Studies Depression Scale (CES-D)^(38)^. Items from the CES-D are included in Appendix B.

## Analytic approach

**Fig. 1:**
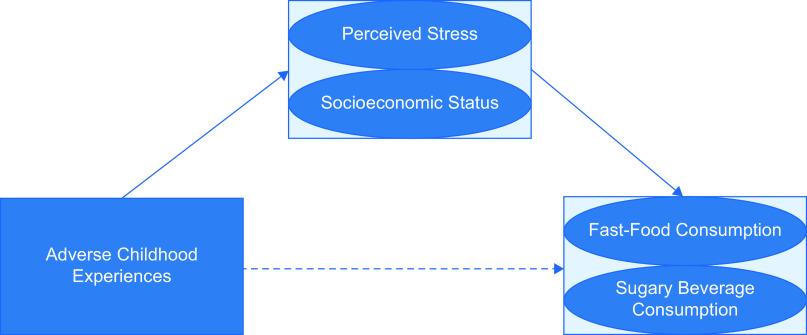
Conceptual mode of the association between adverse childhood experiences and fast-food and sugary beverage consumption

The conceptual model guiding our analysis is presented in Fig. 1. The analysis proceeded in the following steps. To test Aim 1 of the direct relationship between ACE and fast-food consumption and sugary beverage consumption, we presented a series of multinomial logistic regression with ACE as the focal independent variable and levels of fast-food consumption and sugary beverage consumption as the focal dependent variables. Multinomial logistic regression is appropriate, considering the focus of the analysis on differences among categories across the dependent variable^(39)^. Across the analyses, we performed the multinomial logistic regressions in a two-stage process: Model 1 included control variables, and Model 2 included mediating variables for SES and perceived stress at Wave V. To test Aim 2 assessing the indirect effects of SES and perceived stress on levels of fast-food and sugary beverage consumption, we used Karlson–Holm–Breen (KHB) mediation analysis for non-linear models^(40)^.

The KHB method is beneficial in determining the mediation effects in non-linear regression models, such as multinomial logistic regression analyses used in the current study. Specifically, comparing the change in coefficients across non-linear statistical models is challenging because there is a rescaling of the model that occurs whenever a mediator that has an independent effect on the dependent variable is added to a model. The KHB method resolves this issue by adjusting for rescaling and enables a decomposition of the indirect impacts of mediator variables. In addition, a benefit of the KHB approach for the current study is that it allows for a consideration of multiple correlated mediation variables by (1) determining the independent effects of each of the individual mediator variables and (2) calculating whether the change in the independent variable across models following the inclusion of a given mediator variable is greater than chance^(40)^.

Finally, after addressing the main study aims, we conducted a series of supplementary analyses to assess the robustness of the study findings. The supplementary analyses include: (a) changing the classification of the frequency of fast-food and sugary beverage consumption to sextiles instead of quartiles, (b) assessing the outcome variables of fast-food consumption and sugary beverage consumption in the past 7 d using Poisson regression considering the variables were a count distribution and the method is appropriate for determining how ACE predict the frequency of fast-food and sugary beverage consumption in a week^(39)^, (c) using a measure of depressive symptoms from the validated CES-D scale at Wave V^(38)^ instead of perceived stress in the mediation analysis, (d) assessing the association between each specific ACE on fast-food consumption and sugary beverage consumption and (e) examining how the results varied by respondent’s sex and race/ethnicity.

All analyses adjusted for survey weights to account for eligible Wave I respondents who were interviewed at Wave III, IV and V (*gsw1345*). An analysis for multicollinearity indicated that variance inflation factors values were below 2, indicating no significant issues related to multicollinearity.

## Results

Table 1 displays the summary statistics stratified by ACE. Total consumption of fast-food and sugary beverages generally increases in conjunction with accumulating ACE, and this pattern was elevated among those with four or more ACE. Alongside accumulating ACE, there was a reduction in college graduation, household income and an increase in perceived stress.

**Table 1:** Descriptive statistics stratified by adverse childhood experiences (ACE): National Longitudinal Study of Adolescent to Adult Health (*n* 8599)

	ACE – 0		ACE – 1		ACE – 2		ACE – 3		ACE – 4+		
	(*n* 2043)		(*n* 2369)		(*n* 1858)		(*n* 1156)		(*n* 1173)		
Variable	Mean	sd	Mean	sd	Mean	sd	Mean	sd	Mean	sd	*P*
*Fast-food consumption – W5*											
Food fast per week	1·75	1·98	1·87	2·13	2·00	2·30	2·13	2·46	2·32	2·58	<0·001
Fast food – Q1	29·0 %		27·8 %		26·1 %		25·3 %		21·5 %		<0·001
Fast food – Q2	26·7 %		26·8 %		25·1 %		26·0 %		24·1 %		0·248
Fast food – Q3	30·2 %		30·0 %		32·1 %		29·0 %		33·0 %		0·159
Fast food – Q4	14·1 %		15·4 %		16·8 %		19·7 %		21·4 %		<0·001
*Sugary beverage consumption – W5*											
Sugary beverage per week	5·53	6·77	6·09	8·13	6·25	7·61	7·16	10·15	7·80	9·04	<0·001
Sugary beverage – Q1	30·8 %		29·5 %		26·9 %		24·3 %		22·1 %		<0·001
Sugary beverage – Q2	23·7 %		23·9 %		23·8 %		23·2 %		20·8 %		0·450
Sugary beverage – Q3	25·2 %		24·3 %		26·3 %		26·6 %		26·1 %		0·097
Sugary beverage – Q4	20·2 %		22·4 %		23·1 %		26·0 %		31·0 %		<0·001
Age – W5	37·26	1·85	37·41	1·91	37·59	1·83	37·55	1·82	37·60	1·78	0·156
*Race/ethnicity*											<0·001
White	68·2 %		59·6 %		55·3 %		54·9 %		49·3 %		
Black	14·4 %		18·8 %		20·8 %		21·0 %		25·6 %		
Hispanic	10·4 %		12·7 %		15·0 %		15·2 %		15·9 %		
Other race/ethnicity	7·1 %		8·9 %		8·9 %		8·9 %		9·3 %		
Male	44·2 %		42·6 %		41·0 %		39·3 %		39·6 %		0·419
US born	94·1 %		92·6 %		91·9 %		92·3 %		94·5 %		0·571
Married – W5	69·2 %		61·1 %		55·8 %		53·4 %		45·8 %		<0·001
SES – W1	0·48	1·16	0·27	1·27	0·13	1·27	−0·14	1·33	−0·44	1·35	<0·001
*Fruit consumption – W1*											0·018
None	18·6 %		19·5 %		19·6 %		23·1 %		23·9 %		
Once	31·7 %		32·6 %		32·0 %		31·0 %		32·4 %		
More than once	49·6 %		47·9 %		48·5 %		45·9 %		43·6 %		
*Vegetable consumption – W1*											<0·001
None	28·2 %		29·4 %		32·6 %		33·6 %		37·6 %		
Once	40·8 %		41·3 %		38·9 %		37·8 %		34·5 %		
More than once	31·0 %		29·3 %		28·5 %		28·6 %		27·8 %		
Depressive symptoms – W1	1·64	1·77	2·23	2·27	2·69	2·51	3·15	2·82	3·54	2·99	<0·001
College educational attainment – W5	55·0 %		46·9 %		41·5 %		34·5 %		24·3 %		<0·001
Household income ($) – W5	58 088·31	38 117·28	53 635·22	38 064·59	50 079·21	37 941·32	45 224·66	35 516·50	39 688·22	32 657·16	<0·001
Perceived stress – W5	4·26	2·79	4·73	2·87	5·24	3·05	5·68	3·13	5·94	3·21	<0·001

W1, Wave 1; W5, Wave 5; SES, socio-economic status.

*P*-values are derived from *χ*2-test for binary and categorical variables and ANOVA tests for continuous and discrete variables.

Table 2 presents the results of the association between ACE and fast-food consumption. The results in Model 1 showed that relative to those with no ACE exposure, respondents who reported four or more ACE were more likely to report high (relative risk ratio = 1·436, 95 % CI = 1·040, 1·983) fast-food consumption compared with no fast-food consumption. After the mediating variables were included in Model 2, there were no statistically significant relationships at the *P* < 0·05 level. Among the mediating variables, only college educational attainment had a statistically significant association with fast-food consumption in the expected negative direction.

**Table 2:** Multinomial logistic regression of cumulative ACE on fast-food consumption quartiles: National Longitudinal Study of Adolescent to Adult Health (*n* 8599)

	Model 1: with control variables			Model 2: with control variables + mediators		
	Low *v*. none	Medium *v*. none	High *v*. none	Low *v*. none	Medium *v*. none	High *v*. none
Variables	RRR (95 % CI)	RRR (95 % CI)	RRR (95 % CI)	RRR (95 % CI)	RRR (95 % CI)	RRR (95 % CI)
ACE – 1	1·069	1·160	1·060	1·066	1·135	1·030
	0·824–1·387	0·912–1·474	0·800–1·406	0·820–1·384	0·892–1·445	0·776–1·367
ACE – 2	1·005	1·196	1·016	1·006	1·163	0·979
	0·765–1·321	0·933–1·533	0·735–1·403	0·766–1·322	0·903–1·497	0·707–1·355
ACE – 3	1·038	1·074	0·983	1·041	1·029	0·916
	0·742–1·451	0·803–1·436	0·714–1·355	0·741–1·461	0·774–1·368	0·669–1·254
ACE – 4+	1·116	1·222	1·436*	1·104	1·138	1·303
	0·826–1·506	0·906–1·649	1·040–1·983	0·815–1·496	0·845–1·533	0·947–1·794
Age – W5	1·027	1·004	0·994	1·023	1·003	0·991
	0·983–1·072	0·957–1·054	0·933–1·059	0·980–1·069)	0·955–1·052	0·931–1·056
Black	1·164	1·915***	2·245***	1·204	1·972***	2·427***
	0·880–1·538	1·525–2·404	1·628–3·094	0·908–1·595	1·570–2·477	1·750–3·368
Hispanic	1·002	1·349*	1·510	1·005	1·370*	1·535
	0·704–1·426	1·002–1·816	0·981–2·324	0·706–1·431	1·014–1·852	0·994–2·370
Other race/ethnicity	0·898	1·001	1·075	0·902	1·028	1·114
	0·635–1·269	0·683–1·469	0·716–1·616	0·637–1·277	0·705–1·500	0·746–1·663
Male	0·935	1·139	1·562***	0·898	1·094	1·461***
	0·787–1·111	0·976–1·329	1·278–1·909	0·750–1·076	0·933–1·283	1·189–1·796
US born	0·990	1·099	1·132	0·982	1·061	1·098
	0·627–1·563	0·776–1·556	0·643–1·993	0·623–1·548	0·750–1·502	0·622–1·937
Married – W5	1·200	1·165	0·996	1·181	1·244*	1·038
	0·969–1·486	0·993–1·367	0·808–1·228	0·953–1·463	1·050–1·474	0·831–1·296
SES – W1	0·858***	0·771***	0·754***	0·883**	0·829***	0·804***
	0·790–0·933	0·704–0·844	0·681–0·834	0·813–0·958	0·762–0·903	0·728–0·888
Fruit – once – W1	0·952	0·864	0·952	0·963	0·894	0·983
	0·744–1·218	0·688–1·086	0·728–1·245	0·757–1·225	0·711–1·125	0·753–1·285
Fruit – more than once – W1	0·785*	0·663***	0·631**	0·793	0·696**	0·666**
	0·618–0·998	0·529–0·830	0·478–0·832	0·627–1·003	0·555–0·873	0·504–0·881
Vegetables – once – W1	0·890	0·938	0·876	0·903	0·968	0·899
	0·743–1·066	0·773–1·137	0·679–1·130	0·754–1·080	0·797–1·175	0·696–1·160
Vegetables – more than once – W1	0·945	0·984	0·930	0·962	1·024	0·962
	0·762–1·172	0·803–1·205	0·697–1·243	0·777–1·190	0·834–1·258	0·719–1·287
Depressive symptoms – W1	0·977	0·981	1·029	0·979	0·975	1·022
	0·944–1·011	0·949–1·015	0·992–1·069	0·947–1·013	0·942–1·009	0·985–1·060
College educational attainment – W5	–	–	–	0·761**	0·607***	0·515***
	–	–	–	0·635–0·911	0·511–0·720	0·401–0·660
Adjusted household income – W5	–	–	–	1·059	0·986	1·118
	–	–	–	0·935–1·198	0·886–1·097	0·988–1·265
Perceived stress	–	–	–	0·979	1·007	1·030
	–	–	–	0·951–1·008	0·980–1·034	0·996–1·066

ACE, adverse childhood experience; RRR, relative risk ratio; SES, socio-economic status.

Reference categories include 0 ACE, white, no fruit, no vegetables.

* *P* < 0.05.

** *P* < 0.01.

*** *P* < 0.001.

Table 3 presents the results of the relationship between ACE and sugary beverage consumption. The findings showed that net of control variables, respondents with four or more ACE (relative to respondents that reported zero ACE) were more likely to report high sugary beverage intake (relative risk ratio = 1·435, 95 % CI = 1·002, 2·205) compared with the reference category of very low sugary beverage intake. After including the mediating variables in Model 2, there was no statistically significant association between ACE and sugary beverage consumption. Being a college educational attainment was negatively associated with sugary beverage consumption, whereas perceived stress was positively associated with sugary beverage consumption.

**Table 3:** Multinomial logistic regression of cumulative ACE on sugary beverage consumption quartiles: National Longitudinal Study of Adolescent to Adult Health (*n* 8599)

	Model 1: with control variables			Model 2: with control variables + mediators		
	High *v*. very low	Medium *v*. very low	High *v*. very low	Low *v*. very low	Medium *v*. very low	High *v*. very low
Variables	RRR (95 % CI)	RRR (95 % CI)	RRR (95 % CI)	RRR (95 % CI)	RRR (95 % CI)	RRR (95 % CI)
ACE – 1	0·926	0·897	1·090	0·914	0·877	1·045
	0·727–1·179	0·702–1·146	0·862–1·379	0·717–1·165	0·684–1·124	0·819–1·334
ACE – 2	1·069	0·951	1·205	1·039	0·914	1·120
	0·818–1·397	0·762–1·188	0·946–1·535	0·795–1·359	0·730–1·145	0·872–1·438
ACE – 3	0·931	0·890	1·254	0·897	0·843	1·126
	0·674–1·285	0·649–1·221	0·898–1·750	0·650–1·239	0·614–1·157	0·805–1·574
ACE – 4+	0·894	1·035	1·435*	0·851	0·955	1·242
	0·640–1·249	0·769–1·394	1·002–2·055	0·609–1·191	0·707–1·290	0·864–1·786
Age – W5	0·987	0·969	0·967	0·989	0·970	0·968
	0·938–1·039	0·929–1·012	0·921–1·015	0·939–1·041	0·930–1·013	0·922–1·016
Black	2·202***	1·633***	0·894	2·170***	1·610***	0·894
	1·777–2·729	1·294–2·060	0·667–1·198	1·745–2·698	1·271–2·039	0·659–1·212
Hispanic	1·453*	1·300*	0·671**	1·460*	1·311*	0·683**
	1·053–2·006	1·000–1·689	0·506–0·891	1·054–2·022	1·006–1·707	0·513–0·910
Other race/ethnicity	1·428*	1·200	0·782	1·445*	1·229	0·820
	1·023–1·995	0·830–1·735	0·534–1·145	1·038–2·013	0·857–1·761	0·563–1·193
Male	1·247*	1·179	1·680***	1·264*	1·178	1·652***
	1·016–1·530	0·991–1·404	1·406–2·006	1·022–1·562	0·988–1·405	1·375–1·985
US born	1·694**	1·302	1·720*	1·665*	1·259	1·618*
	1·138–2·524	0·883–1·920	1·119–2·643	1·112–2·491	0·848–1·870	1·042–2·511
Married – W5	1·009	0·846*	0·815	1·078	0·939	0·957
	0·844–1·205	0·721–0·992	0·662–1·003	0·898–1·295	0·793–1·111	0·773–1·186
Low SES – W1	0·815***	0·782***	0·733***	0·845***	0·839***	0·820***
	0·753–0·883	0·729–0·839	0·682–0·787	0·781–0·914	0·780–0·902	0·758–0·888
Fruit – once – W1	1·178	1·030	0·913	1·196	1·063	0·965
	0·932–1·489	0·825–1·286	0·738–1·129	0·946–1·512	0·850–1·328	0·781–1·192
Fruit – more than once – W1	0·933	0·713***	0·671***	0·961	0·752**	0·737**
	0·755–1·153	0·585–0·869	0·550–0·820	0·779–1·186	0·616–0·918	0·601–0·904
Vegetables – once – W1	1·069	0·955	0·935	1·078	0·979	0·972
	0·843–1·354	0·761–1·198	0·765–1·143	0·851–1·365	0·781–1·226	0·793–1·192
Vegetables – more than once – W1	1·096	0·951	1·077	1·107	0·981	1·138
	0·860–1·397	0·744–1·215	0·861–1·348	0·870–1·408	0·774–1·245	0·909–1·424
Depressive symptoms – W1	0·971	0·989	1·007	0·962*	0·977	0·988
	0·937–1·005	0·960–1·019	0·972–1·044	0·929–0·997	0·946–1·008	0·953–1·024
College educational attainment – W5	–	–	–	0·913	0·735**	0·517***
	–	–	–	0·746–1·117	0·584–0·924	0·422–0·632
Adjusted household income – W5	–	–	–	0·937	0·909	0·901
	–	–	–	0·826–1·062	0·798–1·036	0·791–1·026
Perceived stress	–	–	–	1·031	1·035*	1·060***
	–	–	–	1·000–1·064	1·003–1·067	1·028–1·094

ACE, adverse childhood experience; RRR, relative risk ratio; SES, socio-economic status.

Reference categories include 0 ACE, white, no fruit, no vegetables.

* *P* < 0.0*5*.

** *P* < 0.01.

*** *P* < 0.001.

Table 4 presents the results of the KHB mediation analysis focusing on the mediating variables with statistically significant associations with the dependent variables. The findings in Panel A showed that having graduated college reduced the association between four or more ACE and high fast-food consumption by 22·69 % (*z*-score = 4·02). The results in Panel B showed that having graduated college reduced the association between 4 or more ACE and sugary beverage consumption by 22·94% (*z*-score = 4·37), and perceived stress reduced the association by 15·43% (*z*-score = 3·20).

**Table 4:** Results of Karlson–Holm–Breen (KHB) mediation analysis of fast-food and sugary beverage consumption: National Longitudinal Study of Adolescent to Adult Health (*n* 8599)

Variables	% Reduction	*z*-score
**Panel A: fast-food consumption**		
College educational attainment	22·69 %	4·02**
**Panel B: Sugary beverage consumption**		
College educational attainment	22·94 %	4·37**
Perceived stress	15·43 %	3·20*

Mediating variables that yielded a statistically significant relationship with the dependent variable in Tables 2 and 3 are mediator variables in the KHB analysis. % Reduction refers to the amount that the mediating variables (college educational attainment or perceived stress) reduce the association between the four + ACE coefficient and the dependent variable (fast-food or sugary beverage consumption).

* *P* < 0·01.

** *P* < 0·001.

### Supplementary analyses

The results in Appendices C–E used quintiles, and Appendices F–H used sextiles of fast-food and sugary beverage consumption. These findings provided similar conclusions to the main results, which classified the variables into quartiles. Appendices I and J provided an analysis using Poisson regression of ACE on the count of fast-food consumption and sugary beverage consumption in the past 7 d. The results offered similar overall patterns showing a positive and significant association between four or more ACE on both dependent variables. The only difference is that the four or more ACE variable retains a positive and statistically significant association after including mediator variables. Next, the analyses were repeated using a measure of depressive symptoms instead of perceived stress, which resulted in similar findings (Appendices K and L). The results in Appendix M assessed the association between each specific ACE on fast-food consumption and sugary beverage consumption, including control variables (Panel A), control variables and mediator variables (Panel B). The results showed few significant associations while accounting for control variables. For instance, community violence was associated with high *v*. no fast-food consumption (relative risk ratio = 1·308, 95 % CI 1·032, 1·332), and emotional abuse was associated with high *v*. very low sugary beverage consumption (relative risk ratio = 1·192, 95 % CI 1·010, 1·408). However, these associations were no longer statistically significant after including mediator variables in Panel B. Finally, ancillary analyses revealed that the main findings of this study did not significantly differ concerning respondents’ sex or race/ethnicity (results available upon request).

## Discussion

The current study aimed to bolster prior research on the relationship between ACE and unhealthy dietary behaviour in adulthood by (1) assessing the relationship between ACE exposure and fast-food and sugary beverage consumption levels and (2) investigating whether the relationship between ACE and higher fast-food and sugary beverage consumption is partly due to higher perceived stress and lower SES. Overall, the current study builds upon existing literature in meaningful ways. To our knowledge, this study is only the second to analyse the connection between ACE and adult dietary behaviour in the USA and is the first to do so with a nationally representative sample in the USA^(9)^. In particular, our focus on fast-food and sugary beverage consumption among those in middle adulthood (aged 33–44 years) provides an essential insight into the relationship between ACE and frequent consumption of high-calorie, nutrient-poor foods in middle adulthood – that is, a period where susceptibility to obesity and nutrition-related chronic disease is heightened^(41)^. Finally, we extend prior research by providing the first analysis of the potential mediating factors in the relationship between ACE and unhealthy dietary behaviour in adulthood.

Our findings corresponding to Aim 1, those assessing the relationship between accumulating ACE exposure and poorer dietary behaviour, showed that four or more ACE are associated with high consumption of fast-food and sugary beverages in adulthood. These findings are consistent with prior literature showing that ACE are related to poorer-quality dietary behaviour in childhood^(10,11)^ and adulthood^(9,26)^, and consistent with research showing that ACE operate in a dose–response manner, such that the concentration of health risk is greatest among those with four or more ACE^(7,8,33)^. Moreover, our supplementary analysis (Appendix M) showed that specific ACE items were not consistently associated with fast-food or sugary beverage consumption, suggesting that rather than being driven by any specific ACE, exposure to a greater dose of accumulating ACE is a driver of higher levels of fast-food or sugary beverage consumption.

Our findings corresponding to Aim 2, those investigating the potential mediating mechanisms in the relationship between ACE and unhealthy dietary behaviour in adulthood, demonstrated that the relationship between ACE and fast-food was mediated by college educational attainment, and the association between ACE and sugary beverage consumption was mediated by college educational attainment and perceived stress. The findings that college education plays a key role in attenuating the relationship between ACE and unhealthy dietary behaviour in adulthood are consistent with research showing college educational attainment to be a uniquely strong factor influencing health behaviours^(24,25)^. Notably, the results also showed that while perceived stress partially mediated the relationship between ACE and sugary beverage consumption, it did not significantly mediate the relationship with fast-food consumption. One possible explanation is that individuals turn to readily available food sources as a coping mechanism in periods of high stress. Many people likely have access to sugar-sweetened beverages in their homes^(42)^. However, accessing fast food requires an individual to travel or order a delivery – both of which will take time before an individual can consume the food item. Thus, in cases of stress, a person may be more likely to turn to sugar-sweetened beverages rather than fast-food out of convenience. Another possibility may be rooted in the demand for quick calories from glucose during periods of stress. For instance, research has found that during periods of acute stress, the energy supply to the brain increases^(43)^, thereby increasing the demand for quick energy sources. Indeed, experimental research has found that stress increases carbohydrate intake to satisfy and meet caloric demands^(44)^. Because sugary beverages are often a faster source of glucose than fast-food, there may be a preference for consuming sugar-sweetened beverages during a period of stress, which may explain why perceived stress was found to mediate the association with sugary beverage consumption but not fast-food consumption.

It is also important to highlight non-significant associations among the household income variable. Notably, while educational attainment was predictive of high fast-food and sugary beverage consumption levels, income was not. Educational attainment may play a more salient role in understanding unhealthy dietary behaviours among adults than income levels. For instance, educational attainment can be pivotal in shaping an individual’s health and nutrition knowledge, attitudes and practices and can impart critical thinking skills and health literacy, enabling individuals to understand better and evaluate nutritional information, health risks and the long-term consequences of their dietary choices^(24,25)^. Conversely, while income undoubtedly influences access to a variety of foods^(45)^, it may not result in the same drive regarding the awareness or prioritisation of healthy eating habits. Overall, the finding of a strong link between educational attainment and dietary behaviour is consistent with a broader literature showing the importance of college educational attainment in promoting positive health behaviours^(24,25)^.

### Limitations and future directions

Some limitations should be considered when interpreting the results. First, at Wave V, only two survey items on dietary behaviour ask about fast-food and sugary beverage consumption. Although fruit and vegetable consumption were inquired at earlier waves, they were not measured at Wave V. While the available dietary measures available at Wave V of the Add Health study tap into unhealthy diet behaviours, it would be beneficial for future research to collect data on a broader range of both healthy and unhealthy dietary patterns individuals engage in, using measures such as comprehensive dietary assessments that include details on fruit and vegetable intake, grains, fibres and added sugars. Second, because the ACE items are asked at different waves with differing reference periods, some ACE are asked about the entirety of childhood and adolescence (i.e. experiences before age 18), and others cover a more limited timeframe (i.e. incidents that occurred by Wave I). Relatedly, because of the gaps between waves (i.e. Wave IV collected in 2008; Wave V in 2016–2018), the outcome variables and mediator variable were both measured at Wave V. While the reference period of the mediating variables for income (past year), educational attainment (highest attainment to date) and perceived stress (past thirty days) temporarily precedes the measure of fast-food and sugary beverage consumption (past 7 d), future longitudinal research that with data collection in shorter time intervals would be helpful to better identify the temporal relationship between how individual-level SES and perceived stress mediate the relationship between ACE and dietary behaviour.

Third, although the study adjusts for a robust set of potential observable variables, any findings should be considered associational rather than causal due to the possibility of omitted variable bias. For instance, accessibility to grocery stores and fast-food restaurants in one’s local area may mediate the findings but were not available at Wave V in the Add Health study. Fourth, we cannot test proximal causal mechanisms between ACE and poor dietary behaviour in this study. ACE-exposed individuals may be prone to poor dietary behaviour as certain unhealthy foods can prompt a release of hormones such as dopamine or serotonin to make themselves feel better. Sixth, while we included a control variable for fruit and vegetable intake at Wave I, it would be helpful for future research with alternative data to better account for patterns of fast-food and sugary beverage consumption throughout childhood and adolescence and into adulthood to capture the possibility of continuity in dietary behaviour among ACE-exposed individuals. Seventh, the study focused on the role of stress, which had clear connections to ACE and dietary behaviour. However, future research can also consider the role of other types of psychological assessments, such as makers of depression or post-traumatic stress. Relatedly, the measure of SES was at the individual level, based on measures of income and educational attainment. However, future research needs to assess the role of neighbourhood-level SES variables, especially because neighbourhood SES may yield independent effects on health-related outcomes^(46)^. It is also important to point out that this study focused on whether perceived stress and individual-level SES mediated the relationship between ACE and fast-food consumption and sugary beverage consumption. However, an important direction for future research would be to assess the complexity of this relationship with alternative methodological approaches, such as mediated-moderation analysis, and investigate potential bidirectional relationships between SES, stress and dietary patterns. Finally, due to the self-report, variables including ACE and fast-food and sugary beverage consumption may be subject to measurement error from recall or social desirability bias. It is also important to note that attrition, patterns of missing data and the use of multiple imputation may have introduced bias in the estimates.

### Study implications

Our research findings also yield implications for future clinical and public health practice and policy that may mitigate the long-term repercussions of ACE exposure and unhealthy dietary behaviours. First, healthcare professionals can screen for ACE and poor dietary behaviours among child and adolescent populations to track the origin of many health-related problems, build a therapeutic relationship with clients and support health promotion and prevention of further diseases and illnesses. Second, scholars have found that helping families set up life routines like participating in family meals can help reduce childhood obesity^(47)^. This strategy may also be adapted to help individuals design daily food practices and ways to mitigate problematic dietary behaviours and promote healthy and balanced eating habits^(48)^. Even so, such programmes may face challenges in homes where individuals are exposed to multiple early-life adversities. Assessing ways to conduct such programmes in trauma-informed manners would be an important avenue of future work. Finally, given the role of college educational attainment and perceived stress in mediating the relationship, a promising avenue may be through promoting resilience training programmes that promote psychological traits around positive emotions, problem-solving and coping skills^(49)^. Likewise, when detecting ACE, it is also important to identify common barriers to academic success and enrol individuals in programmes to improve college readiness^(50)^.

### Conclusion

The findings showed a direct association between four or ACE and high consumption of fast-food and sugary beverages. The results also showed that adjustments for mediating variables, namely college education in the case of fast-food consumption and college educational attainment and perceived stress in the case of sugary beverage consumption, significantly mediated this association. Considering that dietary behaviour is closely connected to health outcomes and the development of chronic diseases that become more prevalent as individuals move through adulthood, these findings can help inform the development of programmatic efforts that mitigate the harm of ACE and provide opportunities to improve the dietary behaviour and overall health of individuals who have exposed to early life trauma.

## Supporting information

Testa et al. supplementary material Testa et al. supplementary material

## Supplementary material

For supplementary material accompanying this paper visit https://doi.org/10.1017/S1368980024000144
