# Supplementary material for: Adverse childhood experiences and unhealthy dietary behaviours in adulthood
Source: Public Health Nutr. 2024 Jan 18;27(1):e40. doi: 10.1017/S1368980024000144 (PMC10882537; doi:10.1017/S1368980024000144)
Supplement: Testa et al. supplementary material [file S1368980024000144sup001.docx]

**Appendix A: Description of ACE Items in the National Longitudinal Study of Adolescent to Adult Health (N = 8,599)**

| **Measure** | **Wave Assessed and Item Description** | **Original Response Range** | **Recoded Response** | **Prevalence** |
| --- | --- | --- | --- | --- |
| *Abuse* |  |  |  |  |
| Emotional Abuse | Wave IV: “Before your 18^th^ birthday, how often did a parent or other adult caregiver say things that really hurt your feelings or made you feel like you were not wanted or loved?” | 0 = never happened, 5 = more than 10 times | 0 = never, 1 = one or more times | 47.5% |
| Physical Abuse | Wave IV: “Before your 18^th^ birthday, how often did a parent or adult caregiver hit you with a fist, kick you, or throw you down on the floor, into a wall, or down stairs?” | 0 = never happened, 5 = more than 10 times | 0 = never, 1 = one or more times | 17.7% |
| Sexual Abuse | Wave III: “Before 6^th^ grade, how often had one of your parents or other adult care-givers touched you in a sexual way, forced you to touch him or her in a sexual way, or forced you to have sexual relations?”  Wave IV: “Before your 18^th^ birthday how often had one of your parents or other adult care-givers touched you in a sexual way, forced you to touch him or her in a sexual way, or forced you to have sexual relations?” | 0 = never happened, 5 = more than 10 times | 0 = never at both waves, 1 = at least once | 7.6% |
| *Household Challenges* |  |  |  |  |
| Community Violence | Wave I: “During the past 12 months, how often did you see someone shoot or stab another person”  Wave I: “During the past 12 months, how often did someone pull a knife or gun on you”  Wave I: “During the past 12 months, how often did someone shoot or stab you”  Wave I: “During the past 12 months, how often did someone cut or stab you” | 0 = never, 2 = more than once | 0 = no exposure, 1 = any exposure | 18.5% |
| Substance Abuse in the Household | Parent Survey: “Does respondent’s biological mother currently have the following health problem: Alcoholism”  Parent Survey: “Does respondent’s biological father currently have the following health problem: Alcoholism”  Wave I: “Are illegal drugs easily available to you in your home” | Parent Survey: Yes/No  Wave I: Yes/No | 0 = no, 1 = yes | 18.1% |
| Suicide exposure | Wave I: “Have any of your family members succeeded in committing suicide in the past 12 months?” | Yes/No | 0 = no, 1 = yes | 0.8% |
| Parental separation or divorce | Parent Survey: “What is your current marital status” | 1 = single, never married; 2 = married; 3 = widowed, 4 = divorced, 5 = separated | 0 = not divorced or separated; 1 = divorced or separated | 17.6% |
| Incarcerated household member | Wave IV: “(Has/did) your (biological mother/  biological father/mother figure/father figure) ever  (spent/spend) time in jail or prison?” | Yes/No | 0 = no parent or guardian incarcerated prior to age 18; 1 = Yes, parent or guardian incarcerated prior to age 18 | 11.8% |
| *Neglect* |  |  |  |  |
| Emotional Neglect | Wave I: (average of relevant items):  Do you agree or disagree with the following statement?   - “Most of the time, your father is warm and loving toward you” - “Most of the time, your mother is warm and loving toward you” - “Overall, you are satisfied with your relationship with your father” - “Overall, you are satisfied with your relationship with your mother” - “You are satisfied with the way your mother and you communicate with each other.” - “You are satisfied with the way your father and you communicate with each other.” | 1 = strongly agree; 5 = strongly disagree | 0 = bottom 80% of low warmth; 1 = top 20% of low warmth | 24.9% |
| Physical Neglect | Wave III: “How often had your parents or other adult care-givers not taken care of your basic needs, such as keeping you clean or providing food or clothing?” | 0 = never happened, 5 = more than 10 times | 0 = never, 1 = one or more times | 10.0% |

**Appendix B: Measurement of the items from the Centers from Epidemiological Studies Depression Scale (CES-D): National Longitudinal Study of Adolescent to Adult Health (N = 8,599)**

| **Items from CES-D** |
| --- |
| How often was the following true during the past week? You felt that you could not shake off the blues even with the help of your family and friends. |
| How often was the following true during the past week? You felt depressed? |
| How often was the following true during the past week? You were happy (reverse coded) |
| How often was the following true during the past week? You felt sad |
| How often was the following true during the past week? You felt Life was not worth living |

Response options include: (0) never or rarely, (1) sometimes, (2) a lot of the time, (3) most of the time or all of the time.

**Appendix C: Multinomial Logistic Regression of Cumulative ACEs on Fast Food Consumption Quintiles: National Longitudinal Study of Adolescent to Adult Health (N = 8,599)**

|  | **Model 1: With Control Variables** | | |  |
| --- | --- | --- | --- | --- |
|  | **Q2 vs. Q1** | **Q3 vs. Q1** | **Q4 vs. Q1** | **Q5 vs. Q1** |
| **Variables** | **RRR**  **(95% CI)** | **RRR**  **(95% CI)** | **RRR**  **(95% CI)** | **RRR**  **(95% CI)** |
| ACEs – 1 | 1.069 | 1.147 | 1.179 | 1.060 |
|  | (0.824 - 1.387) | (0.877 - 1.501) | (0.868 - 1.602) | (0.800 - 1.406) |
| ACEs – 2 | 1.005 | 1.155 | 1.260 | 1.016 |
|  | (0.765 - 1.321) | (0.876 - 1.523) | (0.914 - 1.737) | (0.735 - 1.403) |
| ACEs – 3 | 1.038 | 1.044 | 1.119 | 0.984 |
|  | (0.742 - 1.452) | (0.760 - 1.433) | (0.700 - 1.791) | (0.714 - 1.356) |
| ACEs – 4+ | 1.115 | 1.268 | 1.147 | 1.436* |
|  | (0.826 - 1.506) | (0.921 - 1.745) | (0.747 - 1.760) | (1.039 - 1.983) |
| **Model 2: With Mediator Variables** | | | | |
|  | **Q2 vs. Q1** | **Q3 vs. Q1** | **Q4 vs. Q1** | **Q5 vs. Q1** |
| **Variables** | **RRR**  **(95% CI)** | **RRR**  **(95% CI)** | **RRR**  **(95% CI)** | **RRR**  **(95% CI)** |
| ACEs – 1 | 1.066 | 1.126 | 1.149 | 1.030 |
|  | (0.820 - 1.384) | (0.859 - 1.477) | (0.845 - 1.562) | (0.776 - 1.367) |
| ACEs – 2 | 1.006 | 1.130 | 1.214 | 0.979 |
|  | (0.766 - 1.322) | (0.854 - 1.496) | (0.878 - 1.680) | (0.707 - 1.356) |
| ACEs – 3 | 1.041 | 1.014 | 1.051 | 0.916 |
|  | (0.741 - 1.462) | (0.739 - 1.392) | (0.666 - 1.659) | (0.669 - 1.254) |
| ACEs – 4+ | 1.104 | 1.195 | 1.049 | 1.303 |
|  | (0.815 - 1.496) | (0.868 - 1.644) | (0.686 - 1.603) | (0.947 - 1.794) |

Control variables include: race/ethnicity, sex, U.S. born, married, SES, fruit consumption, vegetable consumption, depressive symptoms. Mediating variables including college graduate, adjusted household income, perceived stress.

*Abbreviations* Q1 = first quantile, Q2 = second quantile, Q3 = third quantile, Q4 = fourth quantile, Q5 = fifth quantile

*** p<0.001, ** p<0.01, * p<0.05

**Appendix D: Multinomial Logistic Regression of Cumulative ACEs on Sugary Beverage Consumption Quintiles: National Longitudinal Study of Adolescent to Adult Health (N = 8,599)**

|  | **Model 1: With Control Variables** | | |  |
| --- | --- | --- | --- | --- |
|  | **Q2 vs. Q1** | **Q3 vs. Q1** | **Q4 vs. Q1** | **Q5 vs. Q1** |
| **Variables** | **RRR**  **(95% CI)** | **RRR**  **(95% CI)** | **RRR**  **(95% CI)** | **RRR**  **(95% CI)** |
| ACEs – 1 | 0.922 | 0.876 | 0.992 | 1.063 |
|  | (0.706 - 1.205) | (0.671 - 1.144) | (0.776 - 1.268) | (0.818 - 1.382) |
| ACEs – 2 | 1.119 | 0.956 | 1.028 | 1.176 |
|  | (0.840 - 1.490) | (0.715 - 1.278) | (0.826 - 1.278) | (0.896 - 1.543) |
| ACEs – 3 | 0.954 | 0.841 | 1.031 | 1.227 |
|  | (0.679 - 1.341) | (0.591 - 1.197) | (0.733 - 1.451) | (0.877 - 1.717) |
| ACEs – 4+ | 0.980 | 0.897 | 1.083 | 1.515* |
|  | (0.695 - 1.382) | (0.634 - 1.269) | (0.762 - 1.540) | (1.049 - 2.186) |
| **Model 2: With Mediator Variables** | | | | |
|  | **Q2 vs. Q1** | **Q3 vs. Q1** | **Q4 vs. Q1** | **Q5 vs. Q1** |
| **Variables** | **RRR**  **(95% CI)** | **RRR**  **(95% CI)** | **RRR**  **(95% CI)** | **RRR**  **(95% CI)** |
| ACEs – 1 | 0.911 | 0.857 | 0.965 | 1.017 |
|  | (0.698 - 1.191) | (0.654 - 1.122) | (0.753 - 1.238) | (0.774 - 1.335) |
| ACEs – 2 | 1.088 | 0.916 | 0.983 | 1.087 |
|  | (0.818 - 1.447) | (0.682 - 1.231) | (0.789 - 1.225) | (0.822 - 1.438) |
| ACEs – 3 | 0.919 | 0.796 | 0.970 | 1.093 |
|  | (0.657 - 1.286) | (0.554 - 1.144) | (0.695 - 1.355) | (0.772 - 1.546) |
| ACEs – 4+ | 0.935 | 0.825 | 0.990 | 1.302 |
|  | (0.663 - 1.317) | (0.577 - 1.180) | (0.698 - 1.404) | (0.895 - 1.892) |

Control variables include: race/ethnicity, sex, U.S. born, married, SES, fruit consumption, vegetable consumption, depressive symptoms. Mediating variables including college graduate, adjusted household income, perceived stress.

*Abbreviations:* Q1 = first quantile, Q2 = second quantile, Q3 = third quantile, Q4 = fourth quantile, Q5 = fifth quantile

*** p<0.001, ** p<0.01, * p<0.05

**Appendix E: Results of KHB Mediation Analysis of Fast-Food and Sugary Beverage Consumption: National Longitudinal Study of Adolescent to Adult Health (N = 8,599)**

| **Panel A: Fast Food Consumption** | | |
| --- | --- | --- |
| **Variables** | **% Reduction** | ***z*-score** |
| College Graduate | 22.70% | 4.02*** |
| **Panel B: Sugary Beverage Consumption** | | |
| **Variables** | **% Reduction** | ***z*-score** |
| College Graduate | 20.48% | 4.21*** |
| Perceived Stress | 14.69% | 3.32** |

*Note:* Mediating variables which yielded a statistically significant relationship with the dependent variable in Appendix C and Appendix D are included as mediator variables in the KHB analysis.

*** p<0.001. ** p<0.01

**Appendix F: Multinomial Logistic Regression of Cumulative ACEs on Fast Food Consumption Sextiles: National Longitudinal Study of Adolescent to Adult Health (N = 8,599)**

|  | **Model 1: With Control Variables** | | |  |  |
| --- | --- | --- | --- | --- | --- |
|  | **Q2 vs. Q1** | **Q3 vs. Q1** | **Q4 vs. Q1** | **Q5 vs. Q1** | **Q6 vs. Q1** |
| **Variables** | **RRR**  **(95% CI)** | **RRR**  **(95% CI)** | **RRR**  **(95% CI)** | **RRR**  **(95% CI)** | **RRR**  **(95% CI)** |
| ACEs – 1 | 1.069 | 1.147 | 1.180 | 0.820 | 1.157 |
|  | (0.824 - 1.387) | (0.877 - 1.501) | (0.869 - 1.603) | (0.547 - 1.230) | (0.691 - 1.936) |
| ACEs – 2 | 1.005 | 1.155 | 1.261 | 0.880 | 1.158 |
|  | (0.765 - 1.321) | (0.876 - 1.523) | (0.915 - 1.737) | (0.584 - 1.328) | (0.699 - 1.919) |
| ACEs – 3 | 1.038 | 1.044 | 1.121 | 0.765 | 0.919 |
|  | (0.742 - 1.451) | (0.760 - 1.434) | (0.701 - 1.792) | (0.447 - 1.311) | (0.515 - 1.639) |
| ACEs – 4+ | 1.115 | 1.268 | 1.148 | 1.014 | 1.819* |
|  | (0.826 - 1.505) | (0.921 - 1.745) | (0.748 - 1.762) | (0.622 - 1.655) | (1.070 - 3.091) |
| **Model 2: With Mediator Variables** | | | | |  |
|  | **Q2 vs. Q1** | **Q3 vs. Q1** | **Q4 vs. Q1** | **Q5 vs. Q1** | **Q6 vs. Q1** |
| **Variables** | **RRR**  **(95% CI)** | **RRR**  **(95% CI)** | **RRR**  **(95% CI)** | **RRR**  **(95% CI)** | **RRR**  **(95% CI)** |
| ACEs – 1 | 1.065 | 1.126 | 1.150 | 0.793 | 1.125 |
|  | (0.820 - 1.384) | (0.859 - 1.477) | (0.846 - 1.563) | (0.529 - 1.189) | (0.671 - 1.887) |
| ACEs – 2 | 1.006 | 1.130 | 1.214 | 0.840 | 1.127 |
|  | (0.766 - 1.322) | (0.854 - 1.496) | (0.878 - 1.680) | (0.553 - 1.276) | (0.684 - 1.857) |
| ACEs – 3 | 1.041 | 1.015 | 1.052 | 0.699 | 0.870 |
|  | (0.741 - 1.461) | (0.740 - 1.392) | (0.667 - 1.660) | (0.408 - 1.196) | (0.490 - 1.543) |
| ACEs – 4+ | 1.103 | 1.195 | 1.050 | 0.905 | 1.664 |
|  | (0.814 - 1.495) | (0.868 - 1.645) | (0.687 - 1.605) | (0.555 - 1.475) | (0.978 - 2.831) |

Control variables include: race/ethnicity, sex, U.S. born, married, SES, fruit consumption, vegetable consumption, depressive symptoms. Mediating variables including college graduate, adjusted household income, perceived stress.

*Abbreviations:* Q1 = first quantile, Q2 = second quantile, Q3 = third quantile, Q4 = fourth quantile, Q5 = fifth quantile, Q6 = sixth quantile

*** p<0.001, ** p<0.01, * p<0.05

**Appendix G: Multinomial Logistic Regression of Cumulative ACEs on Fast Food Consumption Sextiles: National Longitudinal Study of Adolescent to Adult Health (N = 8,599)**

|  | **Model 1: With Control Variables** | | |  |  |
| --- | --- | --- | --- | --- | --- |
|  | **Q2 vs. Q1** | **Q3 vs. Q1** | **Q4 vs. Q1** | **Q5 vs. Q1** | **Q6 vs. Q1** |
| **Variables** | **RRR**  **(95% CI)** | **RRR**  **(95% CI)** | **RRR**  **(95% CI)** | **RRR**  **(95% CI)** | **RRR**  **(95% CI)** |
| ACEs – 1 | 0.963 | 0.940 | 0.901 | 1.164 | 1.069 |
|  | (0.724 - 1.280) | (0.698 - 1.266) | (0.692 - 1.173) | (0.840 - 1.612) | (0.815 - 1.401) |
| ACEs – 2 | 1.160 | 1.031 | 0.981 | 1.318 | 1.214 |
|  | (0.829 - 1.624) | (0.742 - 1.434) | (0.759 - 1.269) | (0.922 - 1.885) | (0.895 - 1.648) |
| ACEs – 3 | 0.732 | 0.916 | 0.812 | 1.202 | 1.122 |
|  | (0.500 - 1.071) | (0.617 - 1.360) | (0.568 - 1.160) | (0.704 - 2.052) | (0.772 - 1.630) |
| ACEs – 4+ | 1.018 | 0.923 | 1.074 | 1.282 | 1.573* |
|  | (0.693 - 1.494) | (0.602 - 1.415) | (0.754 - 1.529) | (0.753 - 2.182) | (1.042 - 2.376) |
| **Model 2: With Mediator Variables** | | | | |  |
|  | **Q2 vs. Q1** | **Q3 vs. Q1** | **Q4 vs. Q1** | **Q5 vs. Q1** | **Q6 vs. Q1** |
| **Variables** | **RRR**  **(95% CI)** | **RRR**  **(95% CI)** | **RRR**  **(95% CI)** | **RRR**  **(95% CI)** | **RRR**  **(95% CI)** |
| ACEs – 1 | 0.961 | 0.923 | 0.881 | 1.121 | 1.023 |
|  | (0.722 - 1.280) | (0.686 - 1.242) | (0.675 - 1.151) | (0.807 - 1.557) | (0.772 - 1.355) |
| ACEs – 2 | 1.148 | 0.997 | 0.943 | 1.236 | 1.124 |
|  | (0.821 - 1.607) | (0.719 - 1.384) | (0.728 - 1.220) | (0.859 - 1.779) | (0.825 - 1.531) |
| ACEs – 3 | 0.724 | 0.875 | 0.769 | 1.100 | 1.001 |
|  | (0.497 - 1.056) | (0.587 - 1.303) | (0.538 - 1.101) | (0.652 - 1.856) | (0.683 - 1.468) |
| ACEs – 4+ | 1.008 | 0.863 | 0.991 | 1.129 | 1.355 |
|  | (0.686 - 1.481) | (0.564 - 1.320) | (0.695 - 1.414) | (0.660 - 1.931) | (0.893 - 2.055) |

Control variables include: race/ethnicity, sex, U.S. born, married, SES, fruit consumption, vegetable consumption, depressive symptoms. Mediating variables including college graduate, adjusted household income, perceived stress.

*Abbreviations:* Q1 = first quantile, Q2 = second quantile, Q3 = third quantile, Q4 = fourth quantile, Q5 = fifth quantile, Q6 = sixth quantile

*** p<0.001, ** p<0.01, * p<0.05

**Appendix H: Results of KHB Mediation Analysis of Fast-Food and Sugary Beverage Consumption: National Longitudinal Study of Adolescent to Adult Health (N = 8,599)**

| **Panel A: Fast Food Consumption** | | |
| --- | --- | --- |
| **Variables** | **% Reduction** | ***z*-score** |
| College Graduate | 12.04% | 2.99** |
| **Panel B: Sugary Beverage Consumption** | | |
| **Variables** | **% Reduction** | ***z*-score** |
| College Graduate | 19.53% | 4.23*** |
| Perceived Stress | 11.96% | 2.95** |

*Note:* Mediating variables which yielded a statistically significant relationship with the dependent variable in Appendix F and Appendix G are included as mediator variables in the KHB analysis.

*** p<0.001, ** p<0.01

**Appendix I: Poisson Regression of Cumulative ACEs on Fast Food Consumption and Sugary Beverage Consumption: : National Longitudinal Study of Adolescent to Adult Health (N = 8,599)**

|  | **Fast-Food Consumption** | | **Sugary Beverage Consumption** | |
| --- | --- | --- | --- | --- |
|  | **Model 1: With Controls** | **Model 2: With Mediators** | **Model 1: With Controls** | **Model 2: With Mediators** |
| **Variables** | **IRR**  **(95% CI)** | **IRR**  **(95% CI)** | **IRR**  **(95% CI)** | **IRR**  **(95% CI)** |
| ACEs – 1 | 1.052 | 1.040 | 1.134* | 1.112 |
|  | (0.953 - 1.161) | (0.942 - 1.148) | (1.009 - 1.275) | (0.987 - 1.252) |
| ACEs – 2 | 1.046 | 1.031 | 1.133* | 1.096 |
|  | (0.936 - 1.168) | (0.923 - 1.152) | (1.011 - 1.268) | (0.975 - 1.232) |
| ACEs – 3 | 1.043 | 1.016 | 1.317** | 1.255** |
|  | (0.936 - 1.162) | (0.915 - 1.129) | (1.111 - 1.560) | (1.064 - 1.480) |
| ACEs – 4+ | 1.197** | 1.158* | 1.347*** | 1.264** |
|  | (1.051 - 1.364) | (1.021 - 1.313) | (1.140 - 1.591) | (1.072 - 1.489) |
| Age – W5 | 0.992 | 0.992 | 0.997 | 0.998 |
|  | (0.973 - 1.012) | (0.972 - 1.012) | (0.976 - 1.018) | (0.977 - 1.018) |
| Black | 1.319*** | 1.343*** | 0.691*** | 0.691*** |
|  | (1.189 - 1.462) | (1.211 - 1.490) | (0.611 - 0.781) | (0.611 - 0.783) |
| Hispanic | 1.140 | 1.144 | 0.764*** | 0.774*** |
|  | (0.989 - 1.315) | (0.992 - 1.318) | (0.660 - 0.883) | (0.670 - 0.894) |
| Other Race/Ethnicity | 1.021 | 1.034 | 0.823* | 0.844 |
|  | (0.892 - 1.167) | (0.909 - 1.177) | (0.682 - 0.993) | (0.703 - 1.013) |
| Male | 1.234*** | 1.209*** | 1.310*** | 1.291*** |
|  | (1.148 - 1.326) | (1.125 - 1.299) | (1.208 - 1.421) | (1.190 - 1.401) |
| U.S. Born | 1.074 | 1.062 | 1.291** | 1.252** |
|  | (0.917 - 1.257) | (0.907 - 1.244) | (1.093 - 1.525) | (1.059 - 1.479) |
| Married – W5 | 0.985 | 1.001 | 0.871** | 0.936 |
|  | (0.911 - 1.064) | (0.923 - 1.086) | (0.790 - 0.960) | (0.844 - 1.038) |
| SES – W1 | 0.926*** | 0.945*** | 0.900*** | 0.947*** |
|  | (0.900 - 0.953) | (0.919 - 0.972) | (0.874 - 0.927) | (0.919 - 0.976) |
| Fruit – Once – W1 | 0.928 | 0.938 | 0.864* | 0.888* |
|  | (0.846 - 1.017) | (0.855 - 1.029) | (0.773 - 0.966) | (0.798 - 0.989) |
| Fruit – More than Once – W1 | 0.816*** | 0.833*** | 0.831** | 0.871* |
|  | (0.744 - 0.896) | (0.759 - 0.915) | (0.745 - 0.928) | (0.782 - 0.970) |
| Vegetables – Once – W1 | 1.001 | 1.009 | 0.987 | 1.006 |
|  | (0.920 - 1.089) | (0.928 - 1.098) | (0.897 - 1.085) | (0.915 - 1.106) |
| Vegetables – More than Once – W1 | 1.038 | 1.050 | 1.045 | 1.075 |
|  | (0.934 - 1.154) | (0.944 - 1.167) | (0.932 - 1.173) | (0.958 - 1.207) |
| Depressive Symptoms – W1 | 1.020* | 1.017* | 1.016 | 1.008 |
|  | (1.003 - 1.037) | (1.001 - 1.034) | (0.994 - 1.038) | (0.986 - 1.031) |
| College Graduate | — | 0.808*** | — | 0.711*** |
|  | — | (0.742 - 0.879) | — | (0.653 - 0.775) |
| Adjusted Household Income – W5 | — | 1.026 | — | 0.950* |
|  | — | (0.986 - 1.067) | — | (0.904 - 0.999) |
| Perceived Stress | — | 1.012 | — | 1.020** |
|  | — | (1.000 - 1.023) | — | (1.006 - 1.035) |

*** p<0.001, ** p<0.01, * p<0.05*; Note:* Reference categories include 0 ACEs, White, No Fruit, No Vegetables.

**Appendix J: Results of KHB Mediation Analysis of Fast-Food and Sugary Beverage Consumption: : National Longitudinal Study of Adolescent to Adult Health (N = 8,599)**

| **Panel A: Fast Food Consumption** | | |
| --- | --- | --- |
| **Variables** | **% Reduction** | ***z*-score** |
| College Graduate | 14.14% | 3.89*** |
| **Panel B: Sugary Beverage Consumption** | | |
| **Variables** | **% Reduction** | ***z*-score** |
| College Graduate | 15.07% | 4.87*** |
| Household Income | 1.24% | 1.11 |
| Perceived Stress | 6.69% | 2.58** |

*Note:* Mediating variables which yielded a statistically significant relationship with the dependent variable in Appendix I are included as mediator variables in the KHB analysis. Poisson regression with KHB mediation is considered experimental and should be interpreted with caution.

*** p<0.001, ** p<0.01,

**Appendix K: Multinomial Logistic Regression of Cumulative ACEs on Sugary Beverage Consumption Quartiles: National Longitudinal Study of Adolescent to Adult Health**

**(N = 8,599)**

|  | **Fast-Food Consumption** | | | **Sugary Beverage Consumption** | | |
| --- | --- | --- | --- | --- | --- | --- |
|  | **Low vs. None** | **Medium vs. None** | **High vs. None** | **Low vs. Very Low** | **Medium vs. Very Low** | **High vs. Very Low** |
| **Variables** | **RRR**  **(95% CI)** | **RRR**  **(95% CI)** | **RRR**  **(95% CI)** | **RRR**  **(95% CI)** | **RRR**  **(95% CI)** | **RRR**  **(95% CI)** |
| ACEs – 1 | 1.068 | 1.139 | 1.030 | 0.913 | 0.882 | 1.041 |
|  | (0.820 - 1.390) | (0.894 - 1.449) | (0.777 - 1.365) | (0.717 - 1.164) | (0.688 - 1.132) | (0.816 - 1.328) |
| ACEs – 2 | 1.009 | 1.170 | 0.984 | 1.041 | 0.926 | 1.119 |
|  | (0.766 - 1.329) | (0.911 - 1.501) | (0.713 - 1.358) | (0.797 - 1.360) | (0.740 - 1.159) | (0.874 - 1.434) |
| ACEs – 3 | 1.040 | 1.039 | 0.928 | 0.903 | 0.862 | 1.139 |
|  | (0.733 - 1.475) | (0.779 - 1.386) | (0.678 - 1.272) | (0.654 - 1.246) | (0.628 - 1.184) | (0.816 - 1.590) |
| ACEs – 4+ | 1.109 | 1.150 | 1.317 | 0.851 | 0.975 | 1.242 |
|  | (0.813 - 1.513) | (0.856 - 1.545) | (0.960 - 1.808) | (0.609 - 1.190) | (0.721 - 1.317) | (0.866 - 1.781) |
| Age – W5 | 1.024 | 1.002 | 0.990 | 0.988 | 0.969 | 0.966 |
|  | (0.980 - 1.070) | (0.955 - 1.052) | (0.930 - 1.054) | (0.938 - 1.040) | (0.928 - 1.011) | (0.920 - 1.014) |
| Black | 1.198 | 1.964*** | 2.428*** | 2.182*** | 1.604*** | 0.903 |
|  | (0.906 - 1.583) | (1.563 - 2.467) | (1.752 - 3.367) | (1.755 - 2.713) | (1.265 - 2.034) | (0.666 - 1.226) |
| Hispanic | 1.002 | 1.368* | 1.536 | 1.462* | 1.308* | 0.686** |
|  | (0.705 - 1.426) | (1.013 - 1.848) | (0.994 - 2.374) | (1.053 - 2.030) | (1.005 - 1.701) | (0.516 - 0.912) |
| Other Race/Ethnicity | 0.900 | 1.026 | 1.110 | 1.449* | 1.227 | 0.819 |
|  | (0.637 - 1.273) | (0.703 - 1.498) | (0.741 - 1.665) | (1.038 - 2.021) | (0.855 - 1.762) | (0.563 - 1.192) |
| Male | 0.904 | 1.092 | 1.452*** | 1.252* | 1.166 | 1.625*** |
|  | (0.753 - 1.084) | (0.931 - 1.282) | (1.180 - 1.786) | (1.014 - 1.546) | (0.977 - 1.391) | (1.354 - 1.951) |
| U.S. Born | 0.981 | 1.061 | 1.096 | 1.666* | 1.260 | 1.614* |
|  | (0.621 - 1.549) | (0.750 - 1.502) | (0.620 - 1.935) | (1.113 - 2.496) | (0.850 - 1.869) | (1.035 - 2.517) |
| Married – W5 | 1.173 | 1.234* | 1.031 | 1.081 | 0.925 | 0.962 |
|  | (0.953 - 1.445) | (1.039 - 1.464) | (0.826 - 1.287) | (0.897 - 1.301) | (0.778 - 1.101) | (0.775 - 1.194) |
| SES – W1 | 0.882** | 0.830*** | 0.806*** | 0.846*** | 0.841*** | 0.822*** |
|  | (0.813 - 0.957) | (0.763 - 0.903) | (0.730 - 0.889) | (0.782 - 0.915) | (0.782 - 0.905) | (0.759 - 0.891) |
| Fruit – Once – W1 | 0.964 | 0.895 | 0.983 | 1.197 | 1.065 | 0.965 |
|  | (0.758 - 1.225) | (0.712 - 1.125) | (0.753 - 1.283) | (0.948 - 1.512) | (0.853 - 1.329) | (0.781 - 1.192) |
| Fruit – More than Once – W1 | 0.795 | 0.695** | 0.662** | 0.958 | 0.747** | 0.732** |
|  | (0.628 - 1.006) | (0.554 - 0.872) | (0.501 - 0.876) | (0.776 - 1.182) | (0.613 - 0.911) | (0.597 - 0.898) |
| Vegetables – Once – W1 | 0.901 | 0.969 | 0.900 | 1.080 | 0.983 | 0.976 |
|  | (0.753 - 1.080) | (0.799 - 1.176) | (0.697 - 1.163) | (0.853 - 1.368) | (0.786 - 1.230) | (0.795 - 1.197) |
| Vegetables – More than Once – W1 | 0.959 | 1.025 | 0.966 | 1.112 | 0.987 | 1.147 |
|  | (0.774 - 1.187) | (0.834 - 1.260) | (0.721 - 1.293) | (0.875 - 1.414) | (0.779 - 1.251) | (0.917 - 1.435) |
| Depressive Symptoms – W1 | 0.980 | 0.976 | 1.024 | 0.963* | 0.980 | 0.989 |
|  | (0.948 - 1.012) | (0.944 - 1.010) | (0.987 - 1.063) | (0.930 - 0.998) | (0.950 - 1.011) | (0.954 - 1.025) |
| College Graduate | 0.762** | 0.606*** | 0.512*** | 0.910 | 0.732** | 0.513*** |
|  | (0.637 - 0.912) | (0.510 - 0.720) | (0.399 - 0.657) | (0.745 - 1.112) | (0.582 - 0.920) | (0.420 - 0.628) |
| Adjusted Household Income – W5 | 1.064 | 0.979 | 1.103 | 0.926 | 0.890 | 0.885 |
|  | (0.943 - 1.201) | (0.883 - 1.086) | (0.975 - 1.248) | (0.817 - 1.050) | (0.783 - 1.012) | (0.778 - 1.006) |
| Depressive Symptoms – W5 | 0.976 | 0.995 | 1.016 | 1.028 | 1.009 | 1.054** |
|  | (0.937 - 1.017) | (0.955 - 1.037) | (0.975 - 1.058) | (0.990 - 1.067) | (0.969 - 1.051) | (1.015 - 1.095) |

*** p<0.001, ** p<0.01, * p<0.05*; Note:* Reference categories include 0 ACEs, White, No Fruit, No Vegetables.

**Appendix L: Results of KHB Mediation Analysis of Fast-Food and Sugary Beverage Consumption: National Longitudinal Study of Adolescent to Adult Health (N = 8,599)**

| **Panel A: Fast Food Consumption** | | |
| --- | --- | --- |
| **Variables** | **% Reduction** | ***z*-score** |
| College Graduate | 22.37% | 4.45*** |
| **Panel B: Sugary Beverage Consumption** | | |
| **Variables** | **% Reduction** | ***z*-score** |
| College Graduate | 23.20% | 4.39*** |
| Depressive Symptoms | 16.73% | 2.85** |

*Note:* Mediating variables which yielded a statistically significant relationship with the dependent variable in Appendix K are included as mediator variables in the KHB analysis. Poisson regression with KHB mediation is considered experimental and should be interpreted with caution.

*** p<0.001, ** p<0.01, * p<0.05
